# Immune-mediated cholangiopathies in children: the need to better understand the pathophysiology for finding the future possible treatment targets

**DOI:** 10.3389/fimmu.2023.1206025

**Published:** 2023-10-20

**Authors:** Alina Grama, Alexandra Mititelu, Claudia Sîrbe, Gabriel Benţa, Tudor Lucian Pop

**Affiliations:** ^1^ 2^nd^Pediatric Discipline, Department of Mother and Child, Iuliu Hatieganu University of Medicine and Pharmacy, Cluj-Napoca, Romania; ^2^ 2^nd^Pediatric Clinic and Center of Expertise in Pediatric Liver Rare Disorders, Emergency Clinical Hospital for Children, Cluj-Napoca, Romania

**Keywords:** immune-mediated cholangiopathies, biliary atresia, sclerosing cholangitis, cellular senescence, autophagy

## Abstract

Cholangiopathies are defined as focal or extensive damage of the bile ducts. According to the pathogenetic mechanism, it may be immune-mediated or due to genetic, infectious, toxic, vascular, and obstructive causes. Their chronic evolution is characterized by inflammation, obstruction of bile flow, cholangiocyte proliferation, and progression toward fibrosis and cirrhosis. Immune-mediated cholangiopathies comprise primary sclerosing cholangitis (PSC), autoimmune cholangitis and IgG4-associated cholangitis in adults and biliary atresia (BA), neonatal sclerosing cholangitis (NSC) in children. The main purpose of this narrative review was to highlight the similarities and differences among immune-mediated cholangiopathies, especially those frequent in children in which cholangiocyte senescence plays a key role (BA, NSC, and PSC). These three entities have many similarities in terms of clinical and histopathological manifestations, and the distinction between them can be hard to achieve. In BA, bile duct destruction occurs due to aggression of the biliary cells due to viral infections or toxins during the intrauterine period or immediately after birth. The consequence is the activation of the immune system leading to severe inflammation and fibrosis of the extrahepatic biliary tract, lumen stenosis, and impairment of the biliary flow. PSC is characterized by inflammation and fibrosis of intra- and extrahepatic bile ducts, leading to secondary biliary cirrhosis. It is a multifactorial disease that occurs because of genetic predisposition [human leukocyte antigen (HLA) and non-HLA haplotypes], autoimmunity (cellular immune response, autoantibodies, association with inflammatory bowel disease), environmental factors (infections or toxic bile), and host factors (intestinal microbiota). NSC seems to be a distinct subgroup of childhood PSC that appears due to the interaction between genetic predisposition (HLA B8 and DR3) and the disruption of the immune system, validated by elevated IgG levels or specific antibodies [antinuclear antibody (ANA), anti-smooth muscle antibody (ASMA)]. Currently, the exact mechanism of immune cholangiopathy is not fully understood, and further data are required to identify individuals at high risk of developing these conditions. A better understanding of the immune mechanisms and pathophysiology of BA, NSC, and PSC will open new perspectives for future treatments and better methods of preventing severe evolution.

## 1 Introduction

**Figure 1:**
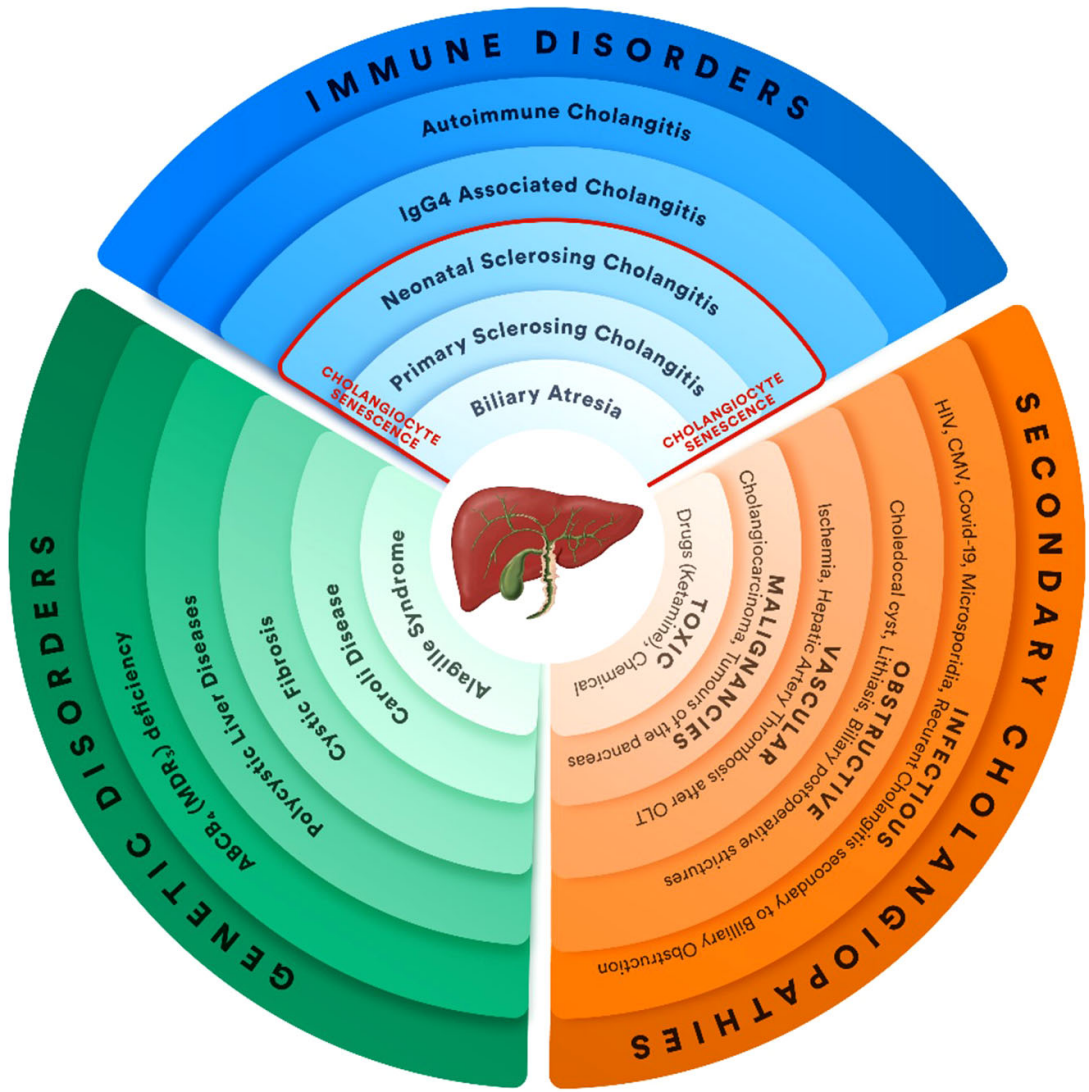
Cholangiopathy classification: immune, genetic disorders, secondary cholangiopathies (OLT, orthotopic liver transplantation) (3–12).

Cholangiopathies are focal or extensive damage of the bile ducts due to genetic, infectious, immune, environmental, or unknown causes. All of these disorders have a chronic evolution characterized by inflammation, biliary fibrosis with the obstruction of bile flow, cholangiocyte proliferation, and progression toward fibrosis and cirrhosis (1–3). According to the pathogenetic mechanism, cholangiopathies are divided into genetic disorders, immune-mediated, infectious, toxic, vascular, and obstructive cholangiopathies. In adults, the most common immune-mediated cholangiopathies are primary biliary cholangitis (PBC), primary sclerosing cholangitis (PSC), autoimmune cholangitis (AC), and IgG4-associated cholangitis (IAC), while, in children, is biliary atresia (BA) (**Figure 1**) (3–12). PBC was named after the first patients presenting with cirrhosis described in 1949 by Dauphinee and Sinclair (13). However, the terminology of the disease changed in 2016 from cirrhosis to cholangitis due to early diagnosis in asymptomatic patients after 2000 (14). PBC is a chronic, autoimmune, progressive liver disorder that is characterized by the presence of antimitochondrial antibodies (AMAs) in serum, cholestasis and specific liver histology (15), while secondary biliary cirrhosis develops after different pathogenic injuries and can result in chronic biliary obstruction (16).

Bile canaliculi surround hepatocytes except for the side next to a sinusoid. The bile canaliculi are composed of hepatocytes’ walls. Bile secreted by hepatocytes circulates through canaliculi toward the center of the liver lamina and flows into hepatic ductules. The ductules fuse into gradually larger ducts. Liver segments are divided by its biliary drainage. The right lobe is branched into anterior (segments V and VIII) and posterior sections (segments VI and VII) (17), and each is divided into superior (VIII and VII) and inferior segments (V and VI). The left lobe is branched into medial (segment IV) and lateral (segments II and III) sections. Biliary segmental ducts are named third-order ducts, sectoral bile ducts are second-order ducts, and the main right and left ducts are considered first-order ducts. The hepatic ducts are located near the portal vein and hepatic artery, which form the portal triad (18).

Immune pathogenesis is operative in all types of immune cholangiopathies leading to chronic inflammation of the bile duct and progressing to cirrhosis (10–12, 19–21). BA, PBC, and PSC are secondary to the increased cholangiocyte senescence process, while AC and IAC are associated with the overproduction of antibodies (19, 20). Cellular senescence, autophagy, and apoptosis are distinct cellular responses to stress. Autophagy plays a significant role in preventing cell damage by maintaining a lysosomal turnover of cellular components. It favors the senescence process and delays cell apoptosis, determining the recovery and repair of normal cell function (19–21). Typically, cellular senescence is produced by combining several factors: DNA deterioration, telomere shortening, and oxidative stress. Senescence is a process in which a cell can no longer replicate or proliferate and is irreversibly arrested in the G1 phase. Senescent cells remain metabolically active and adopt an immunogenic phenotype of pro-inflammatory status (20). PSC, BA, and NSC evolve with severe inflammation, bile duct obstruction, and fibrosis, predominantly extrahepatic biliary ducts in BA or including the entire biliary tree in PSC (2, 21). This narrative review aims to analyze the similarities and differences in the pathological mechanisms of PSC, BA, and NSC, the most frequently encountered immune-mediated cholangiopathies in children.

## 2 Biliary atresia

BA represents the most common cause of cholestasis in infants and the first indication for liver transplantation in children due to its severe and irreversible evolution (21–23). The incidence of BA varies according to geographic region, with 1 to 10–19,000 live births in Europe and North America, while in Asian countries is much higher (1–3,000 live births) (21–24).

### 2.1 The etiopathogenesis of BA

Currently, it is not known precisely what triggers this severe process of obstruction of bile flow. Two major hypotheses underlie the etiology of BA. The first one is that BA appears during organogenesis and the development of the liver, while the second theory supports the hypothesis of an immune disorder (25, 26). Starting from these, BA is classified into embryonal/fetal or syndromic BA (10%–20%) and non-syndromic or acquired/perinatal BA (80%–90%) (24). Embryonal BA is due to defective development of the extrahepatic biliary tract and is often associated with other congenital anomalies such as midline or left-sided liver, asplenia or polysplenia, cardiac malformation (tetralogy of Fallot, dextrocardia, anomalies of the pulmonary or cardiac vessels, atrial and ventricular septal defects), and interrupted inferior vena cava (27, 28). The heterozygous transition of CFC1:c433G>A located in exon 5 was founded in 5 of 10 patients with BA splenic malformation (polysplenia) syndrome, suggesting that genetic predisposition contributes to the disease (29). In those forms of BA, a lack of ductal plate remodeling during bile duct morphogenesis was demonstrated with the persistence of periportal epithelial sleeves (ductal plaque malformation) (30). The existence of cases of BA in the same family and the higher incidence in some regions of the globe (Asian countries) have raised the idea of genetic inheritance (29, 30).

In non-syndromic BA, a combination of factors triggers the inflammatory process (immune system, local and environmental factors). This process appears due to aggression of the biliary cells, mainly due to infections or toxins during the intrauterine period or immediately after birth. The consequence is a severe process of inflammation and fibrosis of the extrahepatic biliary tract, determining lumen stenosis and impairment of the biliary flow (25, 31).

### 2.2 The immune system in BA

The immune system represents perhaps the most important factor in the etiopathogenesis of BA. The liver is an important immunological organ with many properties, such as innate immunity and hematopoiesis in the fetus, immune tolerance, and poor adaptive immune response versus overreactive autoimmunity (32). These immune functions are provided by T lymphocytes, natural killer (NK) cells, macrophages (Kupffer cells), or dendritic cells (DCs) (33). Involvement of the immune system in BA etiopathogenesis was proven by evidence of bile duct damages and inflammatory cells found near them and in cholangiocytes. Also, at the level of the portal spaces, numerous inflammatory cells were revealed: NK cells (CD8), T helper cells (CD4), and histiocytes (CD68). CD4 has more functional subsets, including helper T (Th1, Th2, Th17, and follicular helper), which promotes innate and adaptive immune responses, and the Regulatory T cell (Treg), which suppresses the inflammation resulting from innate and adaptive immunity (34). CD4 marker is typically found on the surface of immune cells such as T helper cells, monocytes, macrophages, and DCs and serves as a co-receptor for the T-cell receptor (TCR). The complex TCR-CD4 binds to distinct regions of the antigen-presenting major histocompatibility complex (MHC) class II molecule. The major role of CD4 is to send signals to other types of immune cells, including cluster of differentiation 8 (CD8) (35). CD8 is a transmembrane glycoprotein co-receptor for the TCR (35). CD8 includes NK cells, the leading cell killer in adaptive immunity, and CD8 Treg cells, which inhibit the activity of Th cells’ immune responses to infection (36, 37) (**Table 1**).

**Table 1:** Pathogenesis and treatment of the most frequent cholangiopathies in children−BA, PSC, NSC.

Characteristics	Biliary atresia	Neonatal sclerosing cholangitis	Primary sclerosing cholangitis
**Genetic predisposition**	- heterozygous transition of CFC1:c433G>A (29)	- autosomal recessive disorder (38) - CLDN1 variant (38) - KMT2D or KDM6A gene variants (39) - HLA B8 and DR3 (40)	- HLA class II (HLA-DR, -DQ, or -DP) (41, 42) - NKG2D gene polymorphisms (43)
**Viruses, bacteria, fungi**	- Cytomegalovirus (44, 45), *Rotavirus*, herpesvirus, adenovirus, reovirus, Epstein–Barr virus (46–49)	- Cytomegalovirus (39)	- intestinal microbiota (50, 51)
**Toxic**	- biliatresone (52) - plants from *Dysphania* (25, 52)	- bile acids (53)	- bile acids (53)
**Anatomy and local factors**	- fetal anatomy (54)	−	- enterohepatic circulation (50)
**Innate and adaptive immunity**	- cholangiocytes (55), macrophages (35), dendritic cells, histiocytes, Kupffer cells (33)	- cholangiocytes (56)	- cholangiocytes, endothelial cells macrophages, dendritic cells and natural killer cells (41, 57, 58)
**Cellular immunity (mediated by T lymphocytes)**	- natural killer cells (CD8+T) (59) - helper T cells (CD4+) (35)	- helper T cells (CD4+) (40)	- natural killer cells (CD8+T) (57) - helper T cells (CD4+) (60)
**Humoral immunity (antibody-mediated immunity)**	- local antibodies against the basal membrane of the biliary epithelium (34, 61) - serum autoantibodies against α-enolase (34, 62)	- antinuclear antibodies (43) -anti-smooth muscle antibody (43)	- Anti-neutrophil cytoplasmic antibodies (63) - antimitochondrial antibodies (41, 63) - anti-thyroperoxidase antibodies (41, 63)
**Maternal microchimerism**	Yes	No	No
**Current therapy**	- Kasai intervention (64) - postoperative steroid administration **-** ursodeoxycholic acid administration - liver transplantation (65)	- liver transplantation (66)	**-** ursodeoxycholic acid administration - liver transplantation (67)

#### 2.2.1 The innate immune system in BA

The innate immune represents the first line of defense against different infections (nonself) or tissue injury (damaged self) (68). In the liver and biliary ducts, this system is represented by the epithelial cells that prevent physical and chemical aggressions. After viral infections, cholangiocyte inflammation represents the immune system’s first response. The process of acute inflammation is initiated by specialized cells (macrophages, DCs, histiocytes, Kupffer cells) that will release inflammatory mediators (cytokines and chemokines) responsible for the clinical signs of inflammation and tissue damage (69). Neutrophils have a major role in the initial inflammatory response to tissue injury. They accumulate near intrahepatic bile ducts in the early phases of viral hepatic injury and produce reactive oxygen species and leukotriene, amplifying cellular destruction and inflammation. The initial immune phase is followed by a second adaptive immune phase mediated by lymphocytes, a process controlled by interleukin 12 (IL-12) (69, 70). Macrophages also play an important role in the pathogenesis of BA. These special cells involved in the detection, phagocytosis, and destruction of different antigens are divided into pro-inflammatory macrophages (M1) and restorative macrophages (M2), with a significant role in tissue regeneration (34). In BA, the balance tilts especially toward pro-inflammatory M1, which, once activated, will produce new chemokines. They will recruit other inflammatory cells producing mediators [IL-1β, IL-6, IL-8, tumor necrosis factor (TNF)-α], thus creating a vicious circle responsible for perpetuating inflammation and liver fibrosis (70, 71). IL-6 and IL-8 (CXCL8) are increased in the serum of patients with BA, indicating ongoing inflammation (71). Macrophages and cholangiocytes mainly produce IL-8. Its role is to recruit inflammatory cells (neutrophils, basophils, T cells), mediate innate immune activation, regulate granulocyte recruitment along the vascular wall and degranulation, and activate them. IL-8 mediates liver injury in BA by promoting bile duct reaction and liver fibrogenesis and induces alpha-smooth muscle actin (α-SMA), a marker responsible for collagen synthesis (72). IL-6 is involved in acute and chronic inflammation and is important in the pathophysiology of graft-versus-host disease (GVHD) and BA (35). The macrophages also release transforming growth factor-beta (TGF-β), which will stimulate hepatic stellate cells (HSCs) to synthesize collagen, thus determining fibrosis and progression to cirrhosis (73). Innate immunity expresses Toll-like receptors (TLRs), a class of proteins responsible for inflammatory response, which recognizes pathogen-associated molecular patterns (PAMPs) and realizes an essential connection between innate and adaptive immunity (73, 74).

#### 2.2.2 The adaptive immune system in BA

Adaptive immunity (acquired immunity) supposes an immune response triggered by repeated exposure to an antigen with two main mechanisms: humoral immunity (antibody-mediated immunity) and cellular immunity (mediated by T cells) (75). Adaptive immunity is the body’s response against non-self-processed peptide antigens or self-antigens when this antigen is presented *via* MHC class I and II molecules (76). Normally, antigens are expressed on the cell surface of the antigen-presenting cell after a viral infection. Helper T cells (CD4) activated through the MHC–antigen complex produce cytokines that promote immune defense against virally infected cells. Most Th0 become Th1, which promotes cell-mediated inflammatory responses by inducing the activation of antigen-presenting cells (CD4), cytotoxic cells (CD8), macrophages, or NK cells (77–79). Some T cells will differentiate into cytotoxic T cells (CD8) that express TCRs involved in recognizing specific antigens. This process occurs in the thymus during the development of immature T cells (80). Once activated, CD8 cells will perpetuate inflammation by producing cytokines (IL-2), chemokines [Macrophage inflammatory protein (MIP)-1α, MIP-1β, Regulated upon Activation, Normal T cell Expressed, and Secreted (RANTES), interferon-gamma (IFN-γ), and TNF-α] and by stimulating the macrophage activation that determines infected cells’ destruction (81, 82). The virus also leads to portal tract inflammation mediated by Th1-cell and periductular inflammation secondary to accumulation of the immune cells with progressive obliteration of the bile ducts and fibrosis (83, 84). With the progression of the inflammatory process, macrophages and DCs will determine activation and proliferation of other Th0 cells, which will turn into Th1 cells after encountering IL-12, IL-18, and interferon type 1 (IFN-α, β, or γ) or Th2 cells after encountering IL-4 (84). Th1 cells produce IL-2, IL-12p70, IL-12p40, IFN-γ, and tumor necrosis factor-beta (TNF-β), while Th2 cells produce IL-4, IL-5, IL-6, and IL-10 (81). Therefore, one of the most important roles of these inflammatory processes in the bile ducts returns to Th1 cells and CD8 T cells, found in over 90% of children with BA (77, 81, 84). Due to this reason, BA has many similarities with GVHD seen after allogeneic hematopoietic-like lymphocytic infiltrate in portal space (Kupffer cells, CD4 T cells, and CD8 T cells), with predominance of CD24+ T helper (Th1) cells and a high number of cell adhesion molecules or human leukocyte antigen (HLA class II) markers (26). All of these processes were described in BA and are not encountered in other cholangiopathies (82). Many other pro-inflammatory cytokines were significantly higher in BA, such as TNF-α, IFN-γ, IL-12, IL-23, IL-32, and IL-33. These will perpetuate the inflammatory cascade and extend bile duct injuries (80). IL-12p40 is a subunit of IL-12 and IL-23, and its role is to promote the migration of macrophages or DCs and is a well-known inducer of the Th1 response (77, 83, 84). The level of IL-12p40 is significantly increased in BA and can predict the success of the Kasai intervention: serum level was higher in children with successful Kasai compared to those in which the intervention failed (64, 78, 79). IL-32 stimulates the synthesis of other pro-inflammatory cytokines, thus perpetuating periductal inflammation (61). In parallel with the high levels of these pro- or anti-inflammatory cytokines, lower levels of growth factors have been found in patients with BA, which reflect inflammation (64). Serum levels of those cytokines may serve as noninvasive biomarkers for the disease progression after surgery and could help to identify patients at high risk for poor outcomes. Regarding humoral immunity in BA, quite a few are known. The presence of antibodies (IgM, IgG) was detected in the basal membrane of the biliary epithelium. Also, the serum of patients with BA seems to contain autoantibodies against α-enolase, a cytoplasmic glycolytic enzyme expressed in various cells, including biliary epithelial cells and hepatocytes (61, 62).

### 2.3 The microchimerism in BA

During the last few years, more studies described the role of maternal microchimerism in BA etiopathogenesis. Microchimerism supposes maternal transplacental bidirectional cell trafficking between the mother and the fetus. This process normally occurs in approximately 40% of pregnancies and continues after birth (62). During normal pregnancy, three types of microchimerism occur: fetal microchimerism (transfer of fetal cells from the fetal circulation into the maternal circulation), maternal microchimerism (transfer of maternal cells into the fetal circulation during pregnancy or parturition), or microchimerism in twins (exchange of cells between the fetuses in the uterus) (55, 85). Maternal microchimerism is supposed to trigger biliary inflammation (27, 28), as maternal alloantigens induce the development of fetal T cells with pro-inflammatory potential and protective immune responses. Fetal T cells with pro-inflammatory potential are born in a tolerogenic environment and form the fetus’s immune system (55, 86). In BA, maternal cells passed to the fetus in the liver become semiallogeneic and persist after birth for a variable period causing insults. The antigen-presenting cells located in the bile duct epithelium, or the vascular endothelium of the portal space, will become the target of maternal effector lymphocytes, thus triggering hepatic and biliary tissue destruction (27). This process was described by Suskind et al. (26), who compared liver tissue derived from children with BA to those with neonatal hepatitis, detecting a high number of maternal cells in the liver of children with BA. Also, Leveque proposed that ductal injuries start in the fetal period with the transfer of maternal chimeric cells and their adherence to bile duct epithelial cells or endothelial cells in the fetus’s liver, causing acute inflammation of bile ducts (87). As a response to maternal antigen, native T fetal cells will differentiate predominantly in Th1, which accumulates in the liver and lymphoid organs and determine chronic inflammation by suppressing Th17 cells, with an important role in maintaining mucosal barriers. The loss of Th17 cell populations at mucosal surfaces determines chronic inflammation and is associated with multiple inflammatory and immune disorders, including BA (88). In neonates, there is a deficit in circulating Tregs in peripheral blood and a dysfunction of them (34). Also, the reduction of lithocholic acid in the gut of patients with BA will decrease Th17 and Th1 (88). As Th1 decreases, Th2 will proliferate, releasing cytokines (IL-4, IL-5, IL-9, IL-13) that stimulate collagen synthesis and fibrosis and are responsible for intrahepatic and extrahepatic bile duct proliferation (44, 88). In 2014, Toshihiro Muraji (27) also demonstrated the presence of a higher number of chimeric maternal cells in the portal area and sinusoids. He elaborated on a few hypotheses explaining the role of chimeric cells in triggering BA (27, 44, 88). The first refers to a primary insult triggered by the migration of chimeric cells in the first semester of pregnancy, along with intrahepatic and extrahepatic duct development (27, 44, 88). Chimeric cells will attach to the portal biliary and endothelial cells from different liver segments. The fetus will develop tolerance against them over time by the production of TGF-β at the level of the lymph nodes. The TGF-β will stimulate Treg fetal synthesis, a subpopulation of T cells, working in time as memory T cells for maternal antigens and causing immunotolerance later in life. Treg cells suppress cytokine production of B, CD4, CD8, and DCs and protect tissues against autoimmunities (27, 45). Various aggressions like cytomegalovirus (CMV) infection can reduce the number and activity of Treg cells determining the loss of immune tolerance and triggering the inflammatory process. Moreover, repeated exposure of the mother’s immune system to these antigens will trigger the immune attack and distort bile duct architecture (44, 45). Exposure to non-inherited maternal antigens (NIMAs) in fetal life and the development of tolerance to the maternal cells (NIMA effect) can be another trigger for bile duct destruction in BA. The last theory is about the injury of the fetal liver determined by maternal lymphocytes during the Kasai intervention (27, 44, 45). Along with this intervention, chimeric cells in the liver become semiallogenes causing GVHD interactions and new injuries of hepatic structures. Complement C4d deposits are present at the level of the endothelial cells in the portal space like renal complexes encountered in renal peritubular capillaries of patients with kidney transplants. This is an important indicator for acute antibody-mediated rejection (AMR) (89, 90). These findings suggest that complement activation led to progressive portal vein damage and stimulated BA fibrogenesis (89, 90).

### 2.4 Environmental factors in BA

Studies in animal models described the important role of environmental factors in BA etiopathogenesis. Viral infections with CMV, *Rotavirus*, reovirus, herpesvirus, adenovirus, and Epstein–Barr virus (EBV) are the leading cause of BA. In 1974, Benjamin Landing elaborated the first theory according to which BA and a few other infantile obstructive cholangiopathies were caused by a viral infection of the liver or biliary ducts (46–49). In non-syndromic BA, the disease often occurs sporadic, with genetic factors having a minor influence, but there is an involvement of the environmental factors and the immune system. Perinatal BA occurs after a viral infection with biliary tropism, which causes the activation of the immune system and determines inflammation and damage to the bile duct, leading to cholangitis associated with ductal and periductal fibrosis (24, 32). The virus must infect neonates, cause viremia, replicate in cholangiocytes, and determine the inflammatory immune response from the host (49). CMV infection is considered an important cause of BA, with a prevalence of 25% (91). Both intrauterine and perinatally CMV infections have been proven to be etiopathogenetic factors in BA (91). CMV injury of cholangiocytes leads to altered immune response, chronic inflammation, and fibrosis of the biliary ducts (92). CMV infection determines the increase of T helper cells (Th1 and Th17) and decrease of regulatory T cells (Th2 and Tregs), causing an exaggerated autoimmune response and bile duct obstruction. CMV also causes the increase of cytokines (IFN-γ, Th1 cytokines, and TNF-α) with inflammation and tissue damage (93). In patients with BA and CMV infection, liver fibrosis is more severe, episodes of cholangitis are more frequent, and progression to cirrhosis is more accelerated compared with patients with BA but without CMV. Moreover, CMV infection seems to be an important prognostic factor in the postoperative evolution of children with BA and Kasai procedure, the evidence of infection predicting an unfavorable prognosis (94). Other authors support that BA is only a genetic abnormality and CMV would not have any role in BA (91).

Reovirus is a member of the family Reoviridae, which determines cholangiocyte infection, followed by necrosis and inflammation in newborn mice used for experimental studies. Reovirus infection also reduces the number of Tregs in the liver, making the bile ducts susceptible to the virus and the immune system (91, 95–97). Reovirus antibodies IgM and IgG have been detected in 55% of cases with BA, but a direct causal relationship has not yet been proven (47, 96).

Another important virus in BA etiopathogenesis is *Rotavirus*, another genus in the family Reoviridae (98). Recent findings on mice indicate that *Rotavirus* induces BA only if the infectious dose is high enough for the virus to escape into the circulation, infecting many cholangiocytes and triggering the immune response (96). An important finding is related to the time of infection. Studies on pregnant female mice infected with *Rotavirus* proved that the infection did not cause BA in the pups, although the viremia was very high. Instead, the postnatal infection can determine Treg cell paucity, disturbing the balance between immune activation and immune tolerance and making the bile ducts susceptible to infection. Another theory postulates that *Rotavirus* infection in the first days of life determines BA due to an immature neonatal murine immune system (47).

Exogenous toxins are possible environmental factors implicated in BA; their role being proven only in animals. Environmental toxins, such as plants from *Dysphania* species, cause biliary damage in Australian newborn lambs or mice. Thus, in 2007, Park et al. noticed that more lambs developed BA after they were fed with *Dysphania* plants found in some arid areas. Also, Michael Pack and Rebecca Wells’s team isolated another plant toxin (isoflavone or biliatresone) that causes extrahepatic bile duct destruction in mammalian cells, supposing the hypothesis that the same process could happen in humans (25, 52).

### 2.5 The local factors in BA

The local factors, cholangiocytes, HSC, or fetal anatomy, are important in BA etiopathogenesis. The main functions of cholangiocytes consist of forming and secreting the primary bile into canaliculi, transport of bile, various ions, solutes, and water across the biliary tree (54, 99, 100). Besides these, cholangiocytes represent the first line of defense of the biliary tract. After viral or bacterial infection, cholangiocytes express a variety of surface innate immune receptors (TLRs), which activate intracellular signaling cascades stimulating the expression of adhesion molecules and the release of cytokines, chemokines, IFN, or other inflammatory mediators (99). IL-8 and IL-15 are the most produced ILs, essential in the chemotaxis and activation of NK cells (59). Cholangiocytes express many cytokines, chemokines, MHC class I and II, CD 44 or TLR-4, and TLR-9 (44, 101). TLRs are transmembrane proteins, expressed on the cell surface, that recognize PAMPs expressed on infectious agents and stimulate the production of cytokines necessary for innate and adaptive immunity (44, 101). TLR recognizes bacterial DNA and distinguishes it from self-DNA (44, 101). Furthermore, in infected cholangiocytes, the release of type 1 IFN-induced apoptosis processes determined cell death and the release of late mediators (34). Hepatic stellate cells (HSCs) determine the progression of liver fibrosis and play an important role in BA evolution (102). HSCs act as antigen-presenting cells and promote NK cell proliferation (102). Once activated, HSCs, together with bone marrow-derived cells, portal fibroblasts, and hepatocytes, become myofibroblasts, which in turn produce extracellular matrix (ECM) composed of glycosaminoglycans and proteoglycans, structural proteins (collagen and elastose), and adhesion proteins, which facilitate binding of the cells to the matrix. In a healthy liver ECM, these components are well balanced. But in BA, excessive liver healing leads to disproportionate deposition of ECM and promotes liver fibrosis (103). The non-syndromic isolated type of BA may result from fetal anatomy (104). There is a defective remodeling of the transition zone between the extrahepatic and intrahepatic bile ducts, called the porta hepatis. Fetal anatomy of the liver could explain the segmental distribution of inflammation and fibrosis of bile ducts. BA starts before the 15th week of gestation when the umbilical vein drains blood flow from the placenta, predominantly in the left branch of the portal vein. That is why, in some cases, the inflammation and fibrosis were more severe on the left lateral segment of the liver (22, 27).

## 3 Neonatal sclerosing cholangitis

Neonatal sclerosing cholangitis (NSC) is a rare autosomal recessive disorder characterized by inflammation and obliterative fibrosis of intrahepatic and often extrahepatic bile ducts, with dilation of preserved segments. Characteristically, the extrahepatic biliary tree is not atretic (38). NSC and BA have many similarities in clinical and histopathological manifestations, and the distinction between the two can be hard to achieve (38). Both have similar features in the first days of life, the particularities becoming more evident during the disease (38). The image scan for the gallbladder is normal in NSC, and histopathology reveals cholangitis at the level of intrahepatic and extrahepatic ducts and sometimes on the pancreatic duct (38, 43, 105). But, similar to BA, the mechanism of tissue damage remains unknown (43).

### 3.1 The etiopathogenesis of NSC

A characteristic of NSC is represented by autoimmune features like antinuclear antibody (ANA) or anti-smooth muscle antibody (ASMA) seropositivity. NSC can be associated with immunodeficiency, inflammatory bowel disease, Langerhans cell histiocytosis, psoriasis, cystic fibrosis, and sickle cell anemia. Also, NSC can be part of two complex syndromes: neonatal ichthyosis-sclerosing cholangitis (NISCH) syndrome and Kabuki syndrome (38). NISCH syndrome is a rare genetic disorder secondary to the CLDN1 variant that determines claudin-1 deficiency and affects hepatic tight junctions (TJs) (38). TJ proteins are localized on the surface of hepatocytes, cholangiocytes, and epidermis and are important for bile secretion, creating a barrier between the blood and bile flow, but also for skin integrity (56). In NISCH syndrome, hepatic variants of TJ molecules increased paracellular permeability for bile acids leading to inflammation and fibrosis of the intra- and extrahepatic bile ducts and biliary cirrhosis (53). Kabuki syndrome is a genetic disorder secondary to KMT2D or KDM6A gene variants (39) characterized by facial dysmorphism, developmental delay, growth hormone deficiency, skeletal anomalies, hypotonia, and congenital heart defect (38).

### 3.2 The immune system in NSC

Even though the name of NSC and PSC is almost similar, the pathogenetic mechanism is different. NSC seems to be a distinct subgroup of childhood PSC, especially the forms that associate specific autoimmune phenomena like elevated IgG levels and a high titer of ANA and ASMA (43). NSC is associated with increased incidence of HLA B8 and DR3, molecules that present antigens to CD4 T helper lymphocytes, initiating the immune response, which denotes the importance of genetic and immunologic factors in the disease etiopathogenesis (40).

## 4 Primary sclerosing cholangitis

PSC is a chronic liver disease characterized by inflammation and fibrosis of intra- and extrahepatic bile ducts, leading to secondary bile cirrhosis. The etiopathogenesis of PSC is not yet fully known, but like BA or NSC, genetic predisposition, infections, and host immunity seem to have significant roles. In addition, an important role in PSC belongs to the intestinal microbiota, with a high association with inflammatory bowel disease (IBD) (40). Up to 75% of people with PSC also have IBD, especially ulcerative colitis (UC) (106). Unlike BA, PSC is relatively rare in children, with an incidence lower than 20%. The disease predominantly affects men aged 30–40 and has a particular geographic distribution (more frequent in Northern Europe than Southern Europe and Asia) (40, 106).

### 4.1 The etiopathogenesis of PSC

In adults, sclerosing cholangitis is divided into PSC with unknown etiology and secondary sclerosing cholangitis (SSC) with a direct causative agent (41, 107). PSC is a multifactorial disease that occurs because of a cycle of immune-mediated cholangiocyte injury leading to fibrosis. Genetic predisposition (associations with HLA and non-HLA haplotypes involved in bile homeostasis and associated with inflammatory regulatory pathways), autoimmunity (involvement of cellular immune response, the presence of various autoantibodies, association of IBD), and environmental factors (infections, selenium or vitamin D deficiency, toxic bile) are the main mechanisms that trigger and maintain inflammation in PSC (41, 107). SSC arises because of the action of a well-known factor that triggers a chronic inflammatory process of bile ducts: infections (CMV, EBV, bacteria, or cryptosporidiosis), drugs (floxuridine), lithiasis, congenital disorders (choledochal cyst, cystic fibrosis), surgical trauma of the bile ducts, ischemia (hepatic artery occlusion after liver transplantation), or malignancies (41, 107).

Unlike BA, genetic predisposition plays a significant role in PSC onset (107). Evidence for genetic susceptibility in PSC is obvious, supported by the different prevalence in certain regions such as Northern and Southern Europe, by a 100 times higher incidence risk for first-degree relatives of PSC patients compared to the general population, of the strong connection with IBD and the presence of autoantibodies (108). PSC occurs because of genetic polymorphisms that determine susceptibility to the disease. The MHC is an important genetic susceptibility locus for the development of PBC. Several HLA molecules are central in the disease coordinating immune responses through the T-cell response (107). Commonly, hepatocytes and cholangiocytes express only HLA class I molecules. Still, in PSC, some HLA class II are aberrantly expressed: HLA-DR on the bile duct epithelium and vascular endothelium, HLA-DP on the bile duct epithelium, but not on the vascular endothelium and HLA-DQ on the bile ducts (109). A more critical role of genetics is linked to cholangiocarcinoma progression: NKG2D gene polymorphisms detected in patients with PSC were associated with a higher risk of cholangiocarcinoma and could identify patients at risk (110). Also, genetic variants of the steroid and xenobiotic receptor (SXR), which protects against bile acid-induced liver injury in mice, are associated with more severe forms of PSC (111).

### 4.2 The immune system in PSC

It is well known that the immune system plays a central role in the etiopathogenesis of PSC, considered an autoimmune disease, even if it does not respect the classic picture of autoimmunity. Thus, it is twice as frequent in men and does not respond to immunosuppressive therapy. However, it presents many features like autoimmunity, such as specific autoantibodies, HLA haplotypes, the association of IBD (70%) or AIH (50%–96%), and the histopathological aspect that describes the presence of a lymphocytic infiltrate in portal areas (42, 112). Mixed inflammatory cells (lymphocytes, plasma cells, and neutrophils) accumulate around the bile ducts. But as the disease progresses, the inflammatory infiltrate is reduced simultaneously with the progressive reduction of bile ducts and the appearance of periductal fibrosis. These changes give the “onion skin” appearance of medium-sized or larger bile ducts, a morphological feature considered characteristic of the disease (113–115).

### 4.3 The innate immune system in PSC

The exposure to toxic bile and the dysregulation of the intestinal microbiota found in PSC patients suggest that innate immunity plays a significant role in PSC etiopathogenesis. The cellular immune response is a primary event in the pathogenesis of PSC, one of the specific features being the abundance of T lymphocytes in the portal spaces, especially CD4, because of the action of different antigens (60). Bacteria, or bacterial constituents (lipopolysaccharide, lipoteichoic acid, peptidoglycans), cross the inflamed intestinal wall and reach the portal circulation (42, 113, 114). Once entered in the portal circulation, these antigens will attract inflammatory cells, such as macrophages, DCs, and NK cells, which will be activated through pattern recognition receptors. All of these activated cells will secrete pro-inflammatory and chemotactic cytokines to maintain the inflammation (57, 58, 116, 117). Also, they will determine MAdCAM-1 expression on portal endothelial cells that plays a central role in recruiting intestinal mucosal lymphocytes to the liver during intestinal inflammation (58). This time, the peripheral blood T-cell level is normal (60). Other authors described a predominance of CD8 T cells or an equal CD4/CD8 ratio. The explanation is related to the areas of the liver where inflammation predominates: CD4 in the portal spaces and CD8 in the lobular areas (57). Cholangiocytes and endothelial cells are important in PSC, as they create a vicious circle leading to inflammation and fibrosis progression (57, 58, 116). Cholangiocytes are the main targets of immune attacks in PSC. After an infection or injury of the biliary ducts, they can secrete pro-inflammatory and pro-fibrotic cytokines and chemokines (IL-1β, IL-6, TNF-α, and IFN-γ) (63, 117–119). Itself, cholangiocytes will be exposed to the action of inflammatory mediators produced by infiltrating inflammatory cells that migrate to the site of the bile duct injury, thus creating a vicious circle that will lead to ductular cholestasis and chronic inflammation (63, 117–119). Bo and his team reported an increased level of TNF-α and IL-1β, both pro-inflammatory cytokines required for activating the innate immune response, mediating the recruitment, activation, and adherence of circulating phagocytic cells (macrophages and neutrophils) and terminating the innate immune response (116, 117). They also described a decreased level of Th-2 mediators (IL-2) in the serum of a patient with PSC (63, 117, 118). Activated cholangiocytes will determine chemotaxis of other immune, mesenchymal, and endothelial cells, all implicated in tissue injury, persistent inflammation, apoptosis, angiogenesis, tissue remodeling processes, and fibrosis (63, 116–120). According to some authors, cholangiocyte activation can also induce the upregulation of HLA molecules and stimulate T and B cells, but more studies are necessary to confirm this hypothesis (121–123). Similar to BA, some T cells will differentiate into CD8 cells that express TCRs with a role in antigen recognition. In PSC, TLRs are found in a large number, in both the biliary and intestinal mucosa. They could be used for predicting the prognosis of the disease due to the correlation with Ludwig fibrosis scores in PSC patients (123). Another similarity with BA is related to cholangiocyte senescence. Normally, biliary epithelial cells have a limited replication capacity in the adult liver. After bile duct injuries, their replication rate increases considerably, thus preventing ductopenia. This property is also valid in liver damage when cholangiocytes become facultative liver stem cells and favor hepatic tissue regeneration (124). In the end stages of PSC, these cells have an increased secretion capacity of numerous cytokines, chemokines, growth factors (IL-6, IL-8, monocyte chemoattractant protein-1, plasminogen activator inhibitor-1, growth factors, and ECM), all this maintaining the inflammation and favoring the progression of fibrosis. Aberrant proliferation and senescence of reactive cholangiocytes in response to a chronic injury underlie new hypotheses regarding the pathogenesis of PSC (125). They also have a high potential for malignant transformation, contributing to the development of cholangiocarcinoma (125). In PSC, blood vascular endothelial cells (BECs) are important in the immune reaction. BECs control the immune response by regulating blood flow and immune cell recruitment by synthesizing cytokines, enzymes, and HLA molecules. Pro-inflammatory cytokines released by BEC stimulate cholangiocytes to secrete chemokines, cytokines, and growth factors that will cause inflammation and fibrosis (107, 113). Like cholangiocytes, BEC does not express HLA class II molecules under normal conditions, but in PSC, these molecules are excessively expressed, especially HLA-DR (109, 115). Some authors support the hypothesis that BECs are antigen-presenting cells, with antibodies attaching to them and stimulating IL-6 synthesis and CD44 expression, a transmembrane glycoprotein involved in cellular aggregation and migration, lymphocyte activation, lymphopoiesis, angiogenesis, and release of cytokines (120). HSCs and portal fibroblasts (PFs) play a major role in hepatic fibrosis by promoting collagen synthesis that begins in the peri-ductular region extending over time throughout the parenchyma (126).

### 4.4 The adaptive immune system in PSC

In PSC, adaptive immunity involves specialized immune cells and antibodies that attack and destroy cholangiocytes. It is a complex process, also found in BA but less in NSC, consisting of responses to specific antigens or self-antigens presented by MHC molecules to antigen-presenting cells to T cells with specialized receptors (TCRs). In PSC, these T cells, especially CD4 cells, infiltrate the portal spaces, while CD8 cells are found in interface hepatitis (110, 129). This finding coincides with a reduced level of peripheral blood CD4+ T cells or defective apoptosis of activated T cells, which may be part of the immune dysregulation observed in patients with PSC (127–129). After the initial response to an antigen, some T cells persist in the body and become long-living memory T that does not require antigen stimulation to proliferate, so they do not need a signal *via* MHC (127–129). Similarities between bacterial antigens from the bowel and self-peptides can trigger the activation of specific T or B cells that can cross-react with self-epitopes responsible for initiating the PSC (130). Activated T cells and macrophages will trigger cholangiocytes’ apoptosis and senescence. The result is immune-mediated damage of the bile ducts, fibrosis, and an increased risk for cholangiocarcinoma (130). Some of the intestinal T lymphocytes will persist as memory cells. They will cross in the portal circulation and, at some point, in the presence of various antigens, can trigger inflammation of the liver and bile ducts. Memory B cells are plasma cells that can produce antibodies for a long time (131). The role of B cells in PSC pathogenesis is not entirely understood. Still, the presence of so many antibodies in the serum of patients with PSC suggests that these cells certainly have a role in the etiopathogenesis of the disease (130). Also, B cells can be detected in the inflammatory infiltrate surrounding bile ducts in PBC, leading to the destruction of intrahepatic bile ducts (132).

The humoral immune response is often encountered in PSC, but in many situations, the meaning of each antibody is not known precisely. Anti-neutrophil cytoplasmic antibodies (ANCAs) are present in the serum of 80% of patients with PSC (117). Other antibodies that can be found in PSC are antimitochondrial (<10%) and anti-thyroperoxidase (7%–16%), and less often, ANA, ASMA, anti-endothelial cell antibody (AECA), or anti-cardiolipin antibody (41, 116). A particular category is represented by antibodies against the pancreatic zymogen granule glycoprotein 2 (GP2) identified in both disorders, PSC and IBD (63). According to some authors, their presence in PSC confirms the involvement of the gut–liver axis in the etiopathogenesis because glycoprotein is commonly expressed in human enterocytes and is not found in hepatobiliary tissue (118).

### 4.5 Environmental factors in PSC

Regarding environmental factors, in PSC, viral infections are not as important a risk factor as in BA. Several viruses have been analyzed, including EBV, CMV, mumps, measles, coxsackie 1-6, and hepatitis B and C viruses, but no association with the disease was detected (133–136). The involvement of toxins in PSC etiopathogenesis was also described. Bile acids (BA) are incriminated in the pathogenesis and progression of chronic fibrosing cholangiopathies, and more importantly, they accelerate carcinogenesis, increasing the risk of cholangiocarcinoma (50, 129, 136–138). Considering that 10%–20% of patients with PSC will develop cholangiocarcinoma, this may be the basis of new therapeutic methods to prevent it (51). Other theories refer to a defect in the hepatobiliary transport system or to arteriosclerosis of the bile duct found especially in those at risk of vascular disease. Altered biliary lipid oxidation and secretion or exposure of endothelial cells to toxic luminal lipid content initiate and favor both the atherosclerosis process and the inflammation in PSC (139).

A significant role in the etiopathogenesis of PSC belongs to the intestinal microbiota. The association between PSC and IBD supports the implications of immune-mediated mechanisms in the initiation and progression of PSC, considered an immune-mediated disease like UC (136). The increased intestinal wall permeability of patients with IBD causes the easy translocation of intestinal bacteria into the portal vein, leading to portal bacteremia and activation of cholangiocytes (136). Kupffer cells in the liver will release chemokines and cytokines attracting other inflammatory cells (macrophages, monocytes, lymphocytes, neutrophils, and fibroblasts) into the portal tracts and peribiliary space, leading to chronic inflammation, periductal fibrosis, obstructive strictures, and finally to secondary biliary cirrhosis (136). At the level of the inflamed intestinal wall but also in the liver, numerous adhesion molecules that attract T cells will be aberrantly expressed (137). These memory T cells accumulated in the inflamed intestine can persist as long-lived memory cells, enter the circulation to the liver, and will trigger the inflammation of portal spaces (50). Clinical studies support the role of intestinal microbiota in PSC: the gut microbiome in patients with PSC and PSC and IBD is different from that in healthy controls and IBD patients only. Also, the frequent association of PSC with IBD demonstrates the role of the gut–liver axis in the disease (50, 51). Experimental studies confirm the implication of the microbiome in PSC pathogeny, proving the involvement of some specific molecules produced by the microbiota in triggering epithelial injury (51, 139).

**Figure 2:**
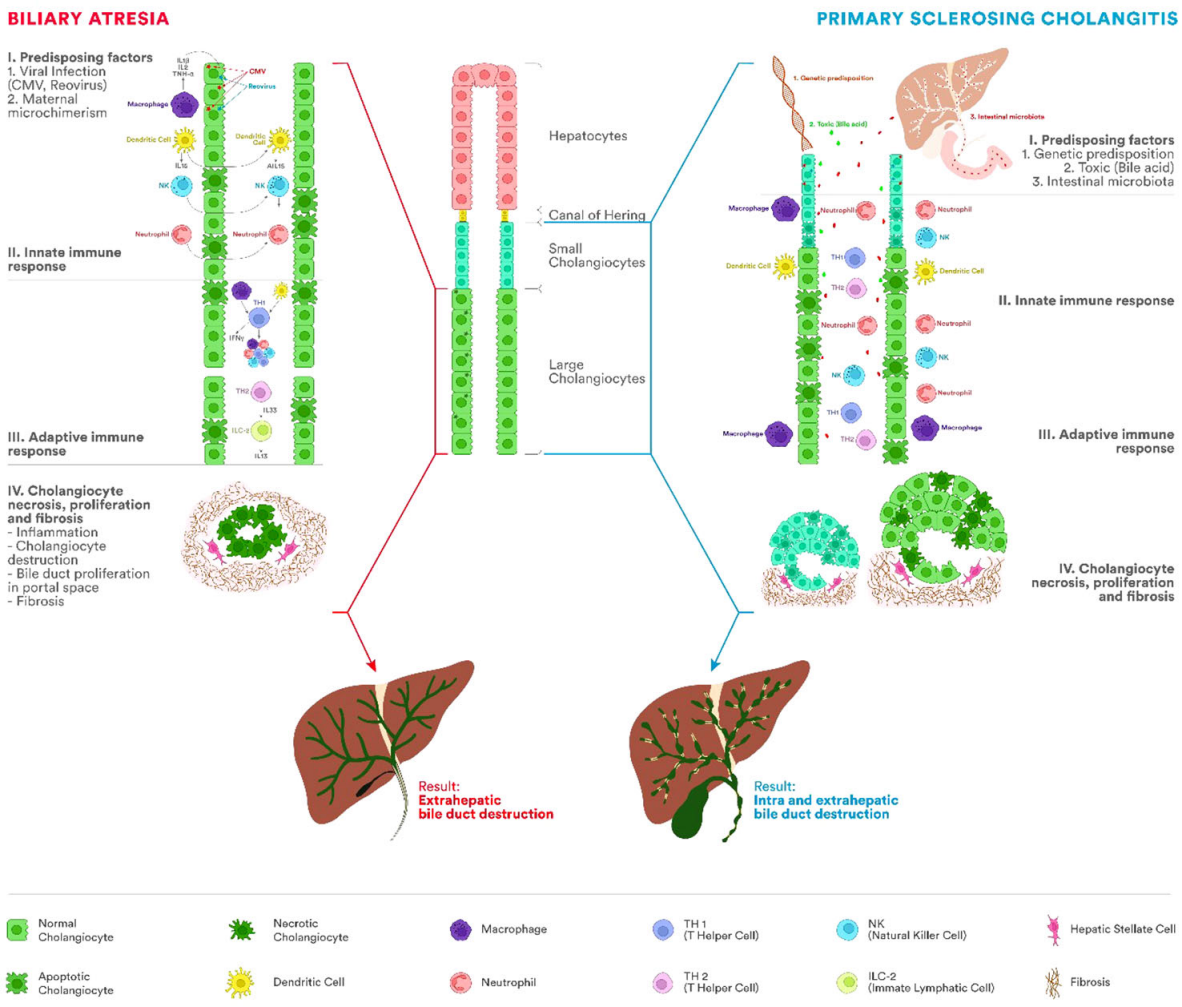
Etiopathogenesis of BA and PSC. The immune system represents one of the most important factors in the etiopathogenesis of BA and PSC, leading to chronic inflammation of the bile duct and progressing to cirrhosis. In BA, the process appears most frequently after viral infections with cytomegalovirus (CMV), *Rotavirus*, or reovirus. PSC occurs in people with a genetic predisposition and different antigens acting as triggers (bile acids, bacteria from the intestinal microbiota). Involvement of innate and adaptive immunity in both diseases has been proven by the presence of innate immune cells, including T lymphocytes, natural killer (NK) cells, macrophages (Kupffer cells), or dendritic cells. In BA, fetal cholangiocyte infection leads to a severe process of inflammation and fibrosis of the extrahepatic biliary tract with stenosis and impairment of the biliary flow. There will be a proliferation of the bile ducts in the portal space, fibrosis, and cirrhosis. Unlike BA, in PSC, the extrahepatic biliary tree is not atretic. In PSC, mixed inflammatory cells (lymphocytes, plasma cells, and neutrophils) are present around the intra- and extrahepatic bile ducts. As the disease progresses, the inflammatory infiltrate decreases simultaneously with the progressive reduction of bile ducts and the appearance of periductal fibrosis and progression to cirrhosis.

Comparing BA and PSC as entities secondary to the increased cholangiocyte senescence process, in **Figure 2**, we summarized the main characteristics of the pathogenesis.

## 5 Conclusions

Despite recent advances in identifying essential aspects regarding the natural history of the most frequent cholangiopathies in children, even with the increasing availability of animal models, a clear and conclusive report concerning disease initiation is still lacking. The most frequently encountered cholangiopathies in children (BA, NSC, and PSC) probably involve genetic predisposition, environmental factors, and the immune system’s dysregulation. Understanding the pathophysiology of different cholangiopathies better is essential to improve the outcome. At present, there is no effective therapy in these entities. Based on the novel findings, future research should aim to find therapies (pharmaceutical or cellular) targeting the disease mechanism at the molecular level. Careful clinical observational studies of clearly defined pediatric patients, together with novel animal models, should further improve our knowledge on the pathogenesis of the most common cholangiopathies in children.

## Author contributions

AG, AM, CS, GB and TP contributed to the conception and design of the study. AG wrote the first draft of the manuscript. AG, AM, CS, GB and TP wrote sections of the manuscript. AG, CS and TP revised the final draft. All authors contributed to the manuscript revision, read, and approved the submitted version.
